# Food provision and healthy eating environments in before school care: an observational study

**DOI:** 10.1017/S1368980025000333

**Published:** 2025-03-21

**Authors:** Andrew J Woods, Yasmine C Probst, Jennifer Norman, Karen Wardle, Sarah T Ryan, Ruth K Crowe, Linda Patel, Megan Hammersley, Kurt Morton, Rebecca M Stanley, Lauren Taylor, Anthony D Okely

**Affiliations:** 1 School of Social Sciences, Faculty of the Arts, Social Sciences and Humanities, University of Wollongong, Wollongong, NSW, Australia; 2 Early Start, Faculty of the Arts, Social Sciences and Humanities, University of Wollongong, Wollongong, NSW, Australia; 3 School of Medical, Indigenous and Health Sciences, Faculty of Science Medicine and Health, University of Wollongong, Wollongong, NSW, Australia; 4 Health Promotion Service, Illawarra Shoalhaven Local Health District, Warrawong, NSW, Australia; 5 Health Promotion Service, South Western Sydney Local Health District, Liverpool, NSW, Australia; 6 Centre for Population Health, NSW Ministry of Health, St Leonards, NSW, Australia

**Keywords:** Out of school hours care, Before school care, Child care, Primary-school children, Healthy eating, Nutrition

## Abstract

**Objective:**

Australian children fall short of national dietary guidelines with only 63 % consuming adequate fruit and 10 % enough vegetables. Before school care operates as part of Out of School Hours Care (OSHC) services and provides opportunities to address poor dietary habits in children. The aim of this study was to describe the food and beverages provided in before school care and to explore how service-level factors influence food provision.

**Design:**

A cross-sectional study was conducted in OSHC services. Services had their before school care visited twice between March and June 2021. Direct observation was used to capture food and beverage provision and child and staff behaviour during breakfast. Interviews with staff collected information on service characteristics. Foods were categorised using the Australian Dietary Guidelines, and frequencies were calculated. Fisher’s exact test was used to compare food provision with service characteristics.

**Setting:**

The before school care of OSHC services in New South Wales, Australia.

**Participants:**

Twenty-five OSHC services.

**Results:**

Fruit was provided on 22 % (*n* 11) of days and vegetables on 12 % (*n* 6). Services with nutrition policies containing specific language on food provision (i.e. measurable) were more likely to provide fruit compared with those with policies using non-specific language (*P*= 0·027). Services that reported receiving training in healthy eating provided more vegetables than those who had not received training (*P*= 0·037).

**Conclusions:**

Before school care can be supported to improve food provision through staff professional development and advocating to regulatory bodies for increased specificity requirements in the nutrition policies of service providers.

A healthy diet is important for the physical and mental well-being of children. The WHO^(1)^ states that the establishment of healthy eating behaviours and optimal nutrition in children decreases the risk of obesity and cardiovascular disease (CVD), supports healthy adulthood and ageing and strengthens learning potential. Dietary risk factors are a leading global public health risk, contributing to 11 million deaths in 2017^(2)^. In Australia, poor dietary intake is the fourth highest risk factor contributing to death and disability combined^(3)^.

During 2020–2021, 37 % of Australian children aged 2–17 years were not meeting the recommended guidelines for fruit intake, and 90 % were not meeting vegetable recommendations^(4)^. Additionally, in the last comprehensive survey of diet in Australian children during 2011–2012, 38 % of children’s daily energy intake was reportedly from low-nutrient and energy-dense discretionary foods^(5)^. With the dietary intake of Australian children falling short of national recommendations, every opportunity should be taken to promote good nutrition and healthy eating.

Out of School Hours Care (OSHC) has been identified as a priority setting for addressing poor dietary habits among Australian children^(6,7)^. In Australia, OSHC offers supervision and care to children aged 5–12 years before and after school and during school holiday periods (vacation care). OSHC services are usually located on school grounds; however, some operate out of day care/pre-schools or community centres. They can either be run by the school or privately, often by local community organisations. With over 4600 OSHC services operating in Australia as of June 2022^(8)^ and just under half a million children attending care on a regular basis^(9)^, OSHC is a widely utilised setting. Food and beverages are usually provided by the service for the children to consume at breakfast (before school care), a snack at afternoon tea (after school care) and sometimes for meals during vacation care. Previous studies have observed the quality of snacks provided in the after school care environment, with US services often failing to meet nutrition policies and predominantly serving high-sugar options^(10)^ and Australian services offering discretionary foods significantly more than vegetables and lean meats^(6)^. Discretionary foods are ‘not an essential or necessary part of healthy dietary patterns’ and are ‘high in kilojoules, saturated fat, added sugars and/or salt’^(11)^.

Understanding the food environment is an important first step for developing meaningful nutrition programmes. Recent after school care intervention studies conducted in the USA have demonstrated a positive effect on services providing and children consuming healthy snacks^(12,13)^. School breakfast programmes in both Denmark and the USA have also resulted in children consuming healthier food options^(14,15)^. Despite these improvements to child eating habits from after school care and school breakfast programmes, limited research has observed the food environment within centre-based before school care. Additionally, Australia does not have a nationally funded school meal programme, with some charitable organisations offering school breakfast programmes on a voluntary basis^(16)^. This is concerning as breakfast is considered important for the cognitive development and academic success of children^(17)^. Understanding the before school care food environment will help inform whether interventions within this setting are also needed and provide a more complete understanding of the food environment within the Australian OSHC sector.

Therefore, this study aimed to (1) describe the foods and beverages provided by OSHC services during before school care and (2) explore how service-level factors influence food provision.

## Methods

### Study design and setting

A cross-sectional observational study was conducted in before school care programmes located in two local health districts of New South Wales (NSW), Australia. The two districts contain a diversity of cultures, socio-economic areas and geographic density^(18,19)^. The University of Wollongong Human Research Ethics Committee approved the study (2019/ETH03798), and a detailed study protocol has been previously published^(20)^.

### Participants

At the commencement of recruitment in November 2020, 267 OSHC providers listed/registered on the Australian Children’s Education and Care Quality Authority website were eligible to participate. Eligibility criteria included services that (a) operated between 06.00 to 09.00 before school; (b) were situated within the geographic bounds of Illawarra Shoalhaven or South Western Sydney Local Health Districts in NSW, Australia; (c) had more than five students enrolled per day; and (d) provided food to children in attendance. All eligible services meeting these criteria were contacted for recruitment via email. An individual service report summarising their findings and recommending improvements was offered as an incentive to participate. Written informed consent was obtained from each OSHC service director, and study information was shared with staff and parents of attending children at least 2 weeks prior to data collection. All staff and children attending the service were eligible to participate in the study, with an opt-out consent approach taken. Information sheets and opt-out consent forms are emailed to families by the service as well as being made available at sign-in areas. If a staff member or child (by their parent/guardian) opted out, they would be disregarded during food environment observations.

The sample size was determined from an unpublished pilot study conducted in the before school care setting^(21)^. As the data in this study were collected as part of a wider study looking at both healthy eating and physical activity in the OSHC setting, the sample size was calculated using physical activity data. This calculation resulted in a required sample of nine OSHC services for 5 % precision. The research team, however, had the capacity to service a larger sample size, and it was determined that up to thirty services could be recruited. Recruitment efforts ceased once this target was met.

### Data collection

Services were visited on two unannounced, non-consecutive days by trained data collectors between March and June 2021. During this data collection period, services were operating normally; however, some had COVID-19 procedures in place which data collectors would need to follow during visits (e.g. signing in and sanitising hands on arrival). The University of Wollongong Human Research Ethics Committee also required a COVID-19 safety plan to be developed and followed during research. This included social distancing from children and staff during visits, wearing face masks and disinfecting all equipment between visits.

Study data were collected and managed using the Research Electronic Data Capture tool (REDCap)^(22,23)^, including the REDCap Mobile Application, hosted at the University of Wollongong, Australia. Data collectors were trained by two of the authors (AW, RC) over a 2-d period. On the first day, participants engaged in theoretical training within a classroom setting, focusing on the study objectives and the tools for data collection. The second day involved an on-site training at a before school care service, during which data collectors had the opportunity to observe typical operations and gain hands-on experience with the data collection tools.

### Food and beverage observation and categorisation

The types of food and beverages provided to children were recorded via direct observation by trained data collectors, following previously published protocols^(20)^. As the purpose of this study was to describe the types of food that before school care services were offering, data were collected on food provision rather than child food consumption. Data were entered by data collectors into a food audit tool developed in REDCap for a family day care study by Kerr *et al.* ^(24)^ and based on a tool created by Kelly *et al.* ^(25)^. Food was entered according to twelve categories: fruit, vegetables, dairy products, non-dairy product drinks, sweet snacks, savoury snacks, breakfast cereals, other grains and cereals, spreads and syrups, meat and alternatives, sauces and condiments and other foods. Photographs were taken of the food provided, as well as the packaging and nutrition labels where available.

Following data collection, the existing categories of the food audit tool were classified into the five food groups according to the Australian Dietary Guidelines: fruit, vegetables, dairy products, grains and lean meats and alternatives^(11)^. Additional groups were added for unsaturated spreads and oils, discretionary foods and discretionary beverages to report foods/beverages that do not fall into the five food groups. Sub-categories of these food groups were added (see Table 1), and food groups were coded dichotomously as offered or not offered^(6)^. To account for the varying nutritional quality of breakfast cereals, additional sub-categories were added for < 15 g/100 g total sugar and ≥ 15 g–< 30 g/100 g total sugar as has been reported previously^(26)^. Breakfast cereals with > 30 g/100 g total sugar (or > 35 g/100 g total sugar if they included added fruit) were classified as discretionary^(27)^. The food classification was conducted by the lead author (AW) and guided by the Australian Health Survey food classification system^(28)^ and the Australian Health Survey Discretionary Food List^(29)^. As food had been previously entered by trained data collectors *a priori* into categories of the food audit tool, one researcher was deemed sufficient for this.

**Table 1.** Food and beverages provided by before school care services over a 2-d observation period (*n* 49)

Food description	Days, *n* (%) food and beverages observed to be offered (*n* 49)	
	*n*	%
Fruit	11	22
Fresh	11	22
Dried	2	4
Canned	0	0
Frozen	0	0
Vegetables	6	12
Fresh	3	6
Canned^†^	3	6
Frozen	0	0
Dairy	49	100
Milk	49	100
Reduced fat/no fat milk	14	29
Full fat milk	38	78
Milk alternatives (e.g. soy and rice milk)	6	12
Cheese (excl. cream cheese)	5	10
Cream cheese spread	26	53
Yoghurt	5	10
Grains	49	100
Wholegrain or higher fibre bread	37	75
Only wholegrain or higher fibre bread^‡^	19	39
Refined grain or lower fibre bread	28	57
Only refined grain or lower fibre bread^§^	10	20
Fruit bread	21	43
Wholegrain or higher fibre breakfast cereal	49	100
Refined grain or lower fibre breakfast cereal	49	100
< 15 % sugar breakfast cereal^*^	48	98
≥ 15 %–< 30 % sugar breakfast cereal^*^	26	53
Oats	10	20
Lean meats and alternatives	4	8
Eggs	2	4
Peanut butter	2	4
Discretionary foods	47	96
Potato products (e.g. hash browns)	1	2
Sweet snacks (e.g. muesli bars)	3	6
Yeast extracts (e.g. Vegemite)	44	90
Sugary products (e.g. jam and honey)	42	86
Discretionary dairy products (e.g. butter)	2	4
Discretionary breakfast cereals (≥30 % sugar)^*^	7	14
Sauces and condiments (e.g. tomato sauce)	3	6
Unsaturated spreads and oils	46	94
Canola oil	1	2
Margarine or table spread	45	92
Beverages		
Water	49	100
100 % fruit juice	2	4

* Based on total sugar per 100 g.

† Baked beans categorised as a vegetable.

‡ Provided wholegrain bread as the only option (i.e. no refined grain bread).

§ Provided refined grain bread as the only option (i.e. no wholegrain bread).

### Food environment measures

The food environment was observed with the aid of two data collection tools. A food and beverage context form was completed at each visit and captured information on child and staff behaviours during breakfast, as well as details on how the meal environment was structured. Data were coded as observed or not observed for the categories: set time for breakfast; children able to serve themselves; restrictions on amount of food; water available as a drink; staff engaged in other duties during mealtime; staff off-task during mealtime (i.e. engaged on their phone); staff eating with children; staff promoting good nutrition (i.e. using positive language when discussing healthy food); staff discouraging good nutrition (i.e. using negative language when discussing healthy food or speaking favourably of unhealthy options); staff providing nutrition education; children preparing breakfast; children distributing food; and children cleaning away breakfast items. On this context form, kitchen facilities were also coded as limited (sink, refrigerator, limited bench space and food storage space), moderate (sink, refrigerator, microwave, moderate bench space and food storage space) or complete (sink, refrigerator, microwave, oven, stove, dishwasher, large bench space and food storage space). This tool and categorisation have been used previously in Australian after school care programmes^(6)^. Inter-observer reliability of this tool has not been tested; however, the tool and categories were covered extensively during data collection training.

A structured interview was conducted in person with each service director/coordinator to capture information on healthy eating policies and practices. The interview was guided by the validated Healthy Afterschool Activity and Nutrition Documentation (HAAND) tool ^(30)^ and took approximately 10 minutes. Responses were recorded in the HAAND tool using the REDCap Mobile Application during the interview. OSHC services are required to have a nutrition policy in Australia by law^(31)^. A copy of this policy was requested in the interview. Policies were assessed for their level of detail and categorised as non-specific (limited detail, talks generally about foods aligning with the Australian Dietary Guidelines) or specific (measurable objectives, discusses serving certain food/beverages)^(6)^. Healthy eating practices captured during the interview included hours of annual staff training, collection of parent/child feedback on breakfast foods and assessment of menu food quality.

Participating OSHC services were also categorised according to their Index of Relative Socio-Economic Advantage and Disadvantage using the Socio-Economic Indexes for Areas published by the Australian Bureau of Statistics^(32)^. Using the percentile ranking of the suburb the service was located in, services were classified into low, medium or high socio-economic tertiles.

### Data analysis

SPSS software (version 24, IBM Corporation) was used to compute descriptive statistics for food and beverages observed to be offered across the two observation days. The mean number of Australian Dietary Guidelines’ five core food groups that were offered was also calculated by averaging the number of groups observed on each visit. Staff behaviours and responses from the interview (i.e. HAAND) were quantified and reported as a percentage of observations and services. Food data were skewed, so a *χ* ^2^ test of independence was used to compare food provision with before school care service characteristics, and a Fisher’s exact test was applied to determine if differences were significant at *P*< 0·05.

## Results

Of the 267 eligible OSHC services, 31 provided consent. As a result of state-wide COVID-19 lockdowns in June 2021, data collection concluded early, and only twenty-five services participated. There were forty-nine observation visits conducted, with each service visited twice, except for one service with a single visit due to COVID-19 lockdowns. Twenty-one (84 %) services were operated by large organisations, and four (16 %) were independently run or part of a long day care service. Ten services (40 %) were in a low socio-economic suburb, six (24 %) in a medium socio-economic suburb and nine services (36 %) in a high socio-economic suburb.

A total of 801 children were in attendance across the observation period. All services offered breakfast to children attending the service. Food was served to children in a family style (i.e. provided multiple options with children allowed to serve themselves) on 49 % (*n* 24) of days. OSHC staff were observed sitting and eating with the children on 29 % (*n* 14) of days, verbally promoting healthy eating on 2 % (*n* 1) and verbally discouraging on 4 % (*n* 2) of days.

Grains (both wholegrains and refined grains) and dairy products were the most commonly served food groups, being provided on 100 % (*n* 49) of observation days. Fruit was provided on 22 % (*n* 11) of days, vegetables on 12 % (*n* 6) and at least one of these two groups provided on 27 % (*n* 13). Lean meats and alternatives were provided on 8 % (*n* 4) of days. Discretionary foods were provided on 96 % (*n* 47) of days with the most common sub-groups being yeast extract spreads (i.e. Vegemite) (90 % of days), sugary products (i.e. jam and honey) (86 % of days) and discretionary breakfast cereals (14 % of days) (Table 1). On average, 2·4 (±0·7) of the five core food groups (i.e. fruit, vegetables, dairy products, grains, lean meats and alternatives) were offered on each observation day.

All services (*n* 25) provided their nutrition policies. Eighteen services (72 %) were part of two larger organisations and used the overarching policy of the organisation; therefore, nine unique policies were reviewed. OSHC services with nutrition policies containing specific language on food provision (i.e. measurable) were significantly more likely to offer fruit during breakfast when compared with those services that used non-specific language regarding food provision (i.e. general statements) within their nutrition policies (*P*= 0·027) (Table 2). OSHC services that reported receiving staff training in healthy eating were observed to provide significantly more vegetables than services that had not received training in healthy eating (*P*= 0·037) (Table 2). Services located in a high socio-economic suburb provided more refined grain bread (*P*= 0·032); however, this was not observed for services providing refined grain bread as their only bread option (Tables 2 and 3).

**Table 2.** Differences in the provision of foods aligning with Australian Dietary Guidelines by service characteristics (*n* 25)

Service characteristics	Fruit	%	Vegetable	%	Dairy	%	Grains	%	Lean meats and alternatives	%	Discretionary	%
Nutrition policy												
Non-specific language (*n* 13)	1	8 %	1	8 %	13	100 %	13	100 %	0	0 %	13	100 %
Specific language (*n* 12)	**6**	**50 %** ^*^	2	17 %	12	100 %	12	100 %	3	25 %	12	100 %
Staff healthy eating training												
No training (*n* 16)	4	25 %	0	0 %	16	100 %	16	100 %	2	13 %	16	100 %
Training (*n* 9)	3	33 %	**3**	**33 %** ^*^	9	100 %	9	100 %	1	11 %	9	100 %
Food quality assessment^†^												
None (*n* 7)	1	14 %	1	14 %	7	100 %	7	100 %	1	14 %	7	100 %
Non-valid (*n* 14)^‡^	5	36 %	1	7 %	14	100 %	14	100 %	1	7 %	14	100 %
Valid (*n* 4)^§^	1	25 %	1	25 %	4	100 %	4	100 %	1	25 %	4	100 %
Breakfast feedback from parents and/or children												
None (*n* 4)	0	0 %	0	0 %	4	100 %	4	100 %	1	25 %	4	100 %
Informal collection (*n* 13)	4	31 %	3	23 %	13	100 %	13	100 %	1	7 %	13	100 %
Formal collection (*n* 8)	3	38 %	0	0 %	8	100 %	8	100 %	1	12·5 %	8	100 %
Service type												
Large organisation (*n* 21)	6	29 %	2	10 %	21	100 %	21	100 %	3	14 %	21	100 %
Independent or long day care (*n* 4)	1	25 %	1	25 %	4	100 %	4	100 %	0	0 %	4	100 %
Kitchen facilities												
Limited/moderate (*n* 10)^||^	4	40 %	2	20 %	10	100 %	10	100 %	2	20 %	10	100 %
Complete (*n* 15)^¶^	3	20 %	1	7 %	15	100 %	15	100 %	1	7 %	15	100 %
Socio-economic area												
Low (*n* 10)	2	20 %	2	20 %	10	100 %	10	100 %	2	20 %	10	100 %
Medium (*n* 6)	2	33 %	0	0 %	6	100 %	6	100 %	1	17 %	6	100 %
High (*n* 9)	3	33 %	1	11 %	9	100 %	9	100 %	0	0 %	9	100 %

Based on a food group being observed in a service on at least one observation day.

*n* = number of before school care services.

* Boldface indicates values are significant *P*< 0·05.

† In comparison with national guidelines.

‡ Self-report.

§ Trained observer or nutrition calculator.

|| Limited (sink, refrigerator, limited bench space and food storage space); moderate (sink, refrigerator, microwave, moderate bench space and food storage space).

¶ Complete (sink, refrigerator, microwave, oven, stove, dishwasher, large bench space and food storage space).

**Table 3.** Differences in the provision of grain foods by service characteristics (*n* 25)

Service characteristics	< 15 % sugar breakfast cereal	%	≥15 %– < 30 % sugar breakfast cereal	%	Discretionary breakfast cereals	%	Wholegrain bread	%	Refined grain bread	%	Only wholegrain bread	%	Only refined grain bread	%
Nutrition policy														
Non-specific language (*n* 13)	13	100 %	8	62 %	5	39 %	12	92 %	8	62 %	8	62 %	2	15 %
Specific language (*n* 12)	12	100 %	6	50 %	1	8 %	10	83 %	9	75 %	4	33 %	5	42 %
Staff training														
No training (*n* 16)	16	100 %	11	69 %	5	31 %	14	88 %	10	63 %	7	44 %	5	31 %
Training (*n* 9)	9	100 %	3	33 %	1	11 %	8	89 %	7	78 %	5	56 %	2	22 %
Food quality assessment^†^														
None (*n* 7)	7	100 %	3	43 %	1	14 %	7	100 %	4	57 %	5	71 %	1	14 %
Non-valid (*n* 14)^‡^	14	100 %	8	57 %	3	21 %	11	79 %	11	79 %	5	36 %	5	36 %
Valid (*n* 4)^§^	4	100 %	3	75 %	2	50 %	4	100 %	2	50 %	2	50 %	1	25 %
Breakfast feedback from parents and/or children														
None (*n* 4)	4	100 %	1	25 %	0	0 %	4	100 %	3	75 %	2	50 %	1	25 %
Informal collection (*n* 13)	13	100 %	7	54 %	3	23 %	11	85 %	9	69 %	6	46 %	4	31 %
Formal collection (*n* 8)	8	100 %	6	75 %	3	38 %	7	88 %	5	63 %	4	50 %	2	25 %
Service type														
Large organisation (*n* 21)	21	100 %	12	57 %	5	24 %	20	95 %	13	62 %	10	48 %	5	24 %
Independent or long day care (*n* 4)	4	100 %	2	50 %	1	25 %	2	50 %	4	100 %	2	50 %	2	50 %
Kitchen facilities														
Limited/moderate (*n* 10)^||^	10	100 %	5	50 %	2	20 %	9	90 %	8	80 %	6	60 %	2	20 %
Complete (*n* 15)^¶^	15	100 %	9	60 %	4	27 %	13	87 %	9	60 %	4	40 %	5	33 %
Socio-economic area														
Low (*n* 10)	10	100 %	4	40 %	3	30 %	9	90 %	5	50 %	5	50 %	2	20 %
Medium (*n* 6)	6	100 %	4	67 %	1	17 %	6	100 %	3	50 %	5	83 %	0	0 %
High (*n* 9)	9	100 %	6	67 %	2	22 %	7	78 %	**9**	**100 %** ^*^	2	22 %	5	56 %

Based on a food group being observed in a service on at least one observation day.

*n* = number of before school care services.

* Boldface indicates values are significant *P*< 0·05.

† In comparison to national guidelines.

‡ Self-report.

§ Trained observer or nutrition calculator.

|| Limited (sink, refrigerator, limited bench space and food storage space); moderate (sink, refrigerator, microwave, moderate bench space and food storage space).

¶ Complete (sink, refrigerator, microwave, oven, stove, dishwasher, large bench space and food storage space).

## Discussion

This cross-sectional study observed the types of food and beverages provided to children (aged 5–12 years) by twenty-five OSHC services in before school care programmes and how these align with the five food groups of the Australian Dietary Guidelines. We also reported on the food environment of these services and explored how these factors influence the provision of different foods. To our knowledge, this is the first study to assess the food environment in Australian before school care services. Breakfast foods served in before school care were primarily grain, dairy products and discretionary foods, with very few services providing fruit, vegetables or lean meats and alternatives. It should also be acknowledged that this study occurred during the COVID-19 pandemic. While OSHC services were operating normally due to limited state-wide restrictions at the time of data collection, caution around the pandemic may have altered staff behaviours (e.g. staff not sitting and eating with children to maintain social distancing).

A lack of fruit and vegetables was observed within the before school care environment, with fruit offered on only 11 observation days (22 %) and vegetables on 6 d (12 %) and at least one of these groups offered on 13 d (27 %). This is concerning as fruit and vegetables are core foods necessary for children’s healthy growth and development^(33)^. Children consuming breakfast at before school care are therefore missing out on an opportunity to contribute to their recommended servings of vegetables each day, which very few Australian children are currently meeting^(4)^. Fruit and vegetable provision in before school care is less than in Australian after school care, with a recent study observing fruit offered to children on 94 % of observation days and vegetables on 44 %^(6)^. While vegetables are often associated with midday and evening meals^(34)^, fruit is commonly provided during the morning in childcare services^(35)^ and should be encouraged in before school care.

A possible means of increasing fruit provision in before school care could be through service policies. This study found that services with a written nutrition policy containing specific measurable language offered significantly more fruit than services whose policies used non-specific language. This is similar to studies conducted in after school programmes in the USA, with interventions involving nutrition policy development with specific measurable language (alongside other intervention components) increasing the provision of fruit^(36)^ and presence of fruit on the menu^(37)^. In Australia, regulations require OSHC services to have a nutrition policy^(31)^; however, there is no requirement for these policies to detail the frequency and categories of foods being provided. As a result, more than half of the services in the study (52 %) had a nutrition policy that made general statements around providing nutritious food and following the Australian Dietary Guidelines. Advocating for OSHC regulating bodies to insist on greater detail in nutrition policies, alongside interventions to support services to develop their policies, could lead to the provision of more fruit offered in OSHC settings.

Further to this, vegetable provision in before school care could be promoted through increased opportunities for staff healthy eating training. We found that services that reported receiving annual healthy eating training provided significantly more vegetables than services that received no training. Similarly, a multi-component intervention that included professional development for after school programme staff in the USA demonstrated increased fruit and vegetable provision in services^(38)^. A recent Australian study found that OSHC staff developed increased confidence and capacity around promoting healthy eating from health promotion professional development^(39)^. Health promotion practitioners should therefore make evidence-based healthy eating training more accessible to staff working in before school care services.

While this study observed discretionary foods being provided on almost all days, a substantial proportion of these were yeast extracts and sugar products used as spreads on toasted bread. Vegemite, jam and honey were the discretionary spreads most frequently observed, and while they are very high in salt and sugar, they are usually consumed in small amounts. There is, however, the possibility of children overconsuming these spreads, so before school care services should be mindful of monitoring intake if they are being provided. Replacing these spreads with healthier alternatives would be preferable and is the recommended advice for childcare services^(40)^.

Our study observed both wholegrain and refined grain breakfast cereals provided on all observation days. It was encouraging to observe wholegrain cereals being offered every morning, as the consumption of wholegrains has been associated with numerous health benefits such as reducing the risk of diabetes and CVD^(41)^. However, providing these wholegrain cereals alongside refined grain cereals, sometimes high in sugar, could reduce the likelihood of children consuming them. An experiment in a US after school programme found that children more often selected unhealthy foods when given a choice among snack offerings^(42)^. With discretionary breakfast cereals (> 30 g/100 g total sugar) observed on 14 % of visits, and refined grain breakfast cereals with > 15 g/100 g total sugar observed on 53 % of visits, children could be forgoing the wholegrain benefits for these sugary options. Before school care would benefit from reducing the refined grain breakfast cereal options to increase the likelihood of children selecting and consuming more wholegrains.

Adults encouraging healthy eating both verbally and through role modelling are considered important for positively shaping children’s eating habits^(43)^. This study observed staff verbally promoting healthy eating on only one observation day and eating with the children on 29 % of observation days. Staff were also observed verbally discouraging healthy eating on two observation days. This finding reveals a need to improve the health-promoting behaviours and knowledge of OSHC staff through methods such as professional development, which has previously led to increased staff promotion of healthy eating in the US after school programmes^(44)^.

### Limitations and strengths

The results of this study should be considered in light of a few limitations. These include: (1) services were located in two local health districts of NSW and may not be representative across NSW or Australia; (2) data collected were on observed food provision so does not reflect actual child consumption; and (3) there is the potential for social desirability bias with service self-reported data. Strengths of this study include: (1) being the first known study to systematically observe food provision and environments in Australian before school care programmes; (2) services were from a diverse geographical and socio-economic area; and (3) food provision was collected observationally rather than with self-report measures.

### Conclusion

This study has provided further evidence to the OSHC literature by observing the food environments of Australian before school care, an area with little to no published research. We found that before school care programmes primarily provide grain, dairy products and discretionary foods to children, with very few fruits, vegetables or lean meat and alternatives served for breakfast. Services with a nutrition policy containing specific measurable language on the types and frequency of foods that should be offered were observed to provide more fruit, and services that reported receiving annual healthy eating training provided more vegetables. There were limited OSHC staff observed verbally promoting healthy eating through conversations during breakfast. The findings of this study reveal a need to improve the variety of food groups being offered in before school care through potential strategies such as targeted professional development for OSHC staff and advocating to regulatory bodies for increased specificity requirements in the nutrition policies of service providers.
